# Statin use and cardiovascular risk factors in diabetic patients developing a first myocardial infarction

**DOI:** 10.1186/s12933-016-0400-y

**Published:** 2016-05-27

**Authors:** Martin Bødtker Mortensen, Imra Kulenovic, Erling Falk

**Affiliations:** Department of Cardiology, Aarhus University Hospital, Palle Juul-Jensens Boulevard, 8200 Aarhus, Denmark

**Keywords:** Prevention, Cardiovascular disease, Diabetes, Statin, Myocardial infarction

## Abstract

**Background:**

The risk for a first myocardial infarction (MI) in people with diabetes has been shown to be as high as the risk for a new MI in non-diabetic patients with a prior MI. Consequently, risk-reducing statin therapy is recommended for nearly all patients with diabetes 40 years of age or older, regardless of cholesterol level. The purpose of this study was to assess the recommended and real-life use of statins for primary prevention of atherosclerotic cardiovascular disease (ASCVD) in diabetic patients who develop ASCVD.

**Methods:**

In a cross-sectional multicenter study of consecutive patients without previous ASCVD hospitalized with a first MI in 2010–2012, we obtained information on diabetic status, statin use, and cardiovascular risk factors prior to MI.

**Results:**

The study population consisted of 1622 patients with first MI (63 % men), 228 of whom had known diabetes before MI. All but three of the diabetic patients were ≥40 years of age. Diabetic patients were older (70 vs 68, p = 0.006), were more often women (43 vs 36 %, p = 0.05) and had a higher prevalence of statin use (47 vs 11 %, p < 0.001) compared with non-diabetic patients. Despite a high risk factor burden, the majority (53 %) of patients with known diabetes was not treated with statins before MI, and there was no relationship between the number of high-risk markers and statin use. Nearly all diabetic patients not treated with statins before first MI had at least one marker of very high cardiovascular risk, including hypertension (71 %), current smoking (37 %), and nephropathy (33 %).

**Conclusions:**

Primary prevention with statins had been initiated in less than half of diabetic patients destined for a first MI, despite the presence of one or more markers of very high cardiovascular risk in nearly all. These results highlight an urgent need for optimizing statin therapy and global risk factor control in diabetic patients.

## Background

It has been known for decades that diabetes is a powerful risk factor for atherosclerotic cardiovascular disease (ASCVD) [1]. Nevertheless, it came as a surprise when a study published in 1998 indicated that the risk for a first myocardial infarction (MI) in people with type 2 diabetes was as high as the risk for a new MI in non-diabetic patients with prior MI [2]. Consequently, the American NCEP (National Cholesterol Education Program) clinical guidelines defined diabetes as a coronary heart disease (CHD) risk equivalent with similar intensity and goal of cholesterol-lowering therapy as in patients with known CHD [3]. Similarly, in 2003, the European guidelines on ASCVD prevention defined diabetes type 2 and diabetes type 1 with microalbuminuria as high-risk conditions and recommended similar treatment goals for diabetic patients without ASCVD and nondiabetic patients with established ASCVD [4]. Today, risk-reducing statin therapy is recommended for nearly all patients with diabetes 40 years of age or older, regardless of cholesterol level [5–11].

To what extent this recommendation is followed in routine clinical practice is not known. Therefore, to address this question, we assessed the uptake of primary prevention with statins in contemporary and consecutive diabetic patients hospitalized with MI as first manifestation of ASCVD.

## Methods

We identified consecutive patients with a first MI without prior ASCVD (hereafter just called first MI) admitted to four hospitals in Denmark in 2010 through 2012 (Aarhus University Hospital and the Regional Hospitals in Randers, Herning and Horsens). From the medical records we collected information about known diabetes treated with diet or drugs (~9 % were type 1 diabetes), use of statins and anti-hypertensive agents, and cardiovascular risk factor burden before hospitalization for first MI. The universal definition of MI is implemented in Denmark, requiring clinical evidence of myocardial ischemia together with elevated biomarkers reflecting myocardial necrosis [12]. The traditional cardiovascular risk factors [age, sex, smoking status, total cholesterol, low-density lipoprotein cholesterol (LDL-C), high-density lipoprotein cholesterol (HDL-C), and systolic blood pressure] were assessed as previously described [13]. Plasma lipid values were obtained within 24 h after admission and/or available from a prior contact with the health care system. The blood pressure used for risk estimation was obtained prior to admission (if hospitalized previous year) or after recovery from MI (before hospital discharge or at first visit to the rehabilitation clinic). Hypertension was defined as SBP >140 mmHg and/or use of anti-hypertensive agents at admission.

### Risk assessment

In 2003, the European Society of Cardiology (ESC) guidelines on ASCVD prevention introduced a new multifactorial risk assessment model for use in the primary prevention of ASCVD in people 40–65 years of age without diabetes, SCORE (Systematic Coronary Risk Evaluation) [4]. Patients with diabetes were classified as high-risk, regardless of other cardiovascular risk factors, and treatment decisions should in principle not be based on SCORE. However, it was suggested that SCORE could be used for a rough assessment of cardiovascular risk in diabetic patients, recognizing that the real risk would be at least twice as high in diabetic men and up to four times higher in diabetic women as that given by SCORE [14]. A more recent re-analysis of the SCORE database indicated that the impact of diabetes on risk might be even greater, with relative risks of ~5 in women and ~3 in men [5, 6, 15]. With this in mind, we calculated SCORE in the total population and estimated also the real risk in patients with diabetes by multiplying the SCORE risk by 5 in women and 3 in men.

The SCORE predictors used to calculate the 10-year risk for fatal CVD include sex, age, smoking status, total cholesterol and systolic blood pressure [4–6]. We used the published SCORE equations [14] to calculate the 10-year risk and capped the age-related risk at age 65 in agreement with the assumptions and complying with clinical practice [4–6]. SCORE was created for use in non-diabetic people 40–65 years of age [14], risk charts are available only for people in this age range [4–6], and the age-related risk is capped at age 65 in the online risk calculator, *HeartScore* [16]. We used the low-risk SCORE equations, as recommended for Denmark in the most recent ESC guideline [6]. In patients without diabetes, statin therapy is indicated or should be considered if SCORE is ≥5 %, defined as high (5–10 %) or very-high (≥10 %) risk [6].

### Primary prevention with statins in diabetes

In diabetic patients, primary prevention with statins is recommended or should be considered in nearly all ≥40 years of age, with the intensity of therapy depending on the presence of other cardiovascular risk factors and end-organ damage [5–7]. Diabetes (type 1 or type 2) with ≥1 cardiovascular risk factor and/or end-organ damage is classified as very high risk, corresponding to SCORE ≥10 % and with a class I recommendation for statin therapy similar to patients with documented ASCVD [5–7]. The national Danish guidelines follow these European guidelines [17–22].

The study was approved by the Danish Data Protection Agency (Reference: 2007-58-0010, int. ref: 1-16-02-46-12). Registry studies do not require ethical approval in Denmark.

### Statistical analysis

Statistical analysis was performed using Stata version 13.1 SE (StataCorp LP, College Station, TX, USA). Baseline characteristics were compared with Student’s t test, Mann–Whitney test or Fisher’s exact test (categorical variables). The 10-year risk of fatal CVD was calculated for each patient using the low-risk SCORE algorithms [14]. We used logistic regression analyses to assess the association between risk factors and use of statins in diabetic patients before MI. These results are presented as odds ratio (OR) with 95 % confidence intervals (CI).

## Results

Among 1632 consecutive patients with first MI, information about diabetes and/or statin use before MI was available in all but ten patients (study population, Table 1). The majority (63 %) were men. They were ~7 years younger than women and had a higher predicted risk estimated by SCORE (mean 4.6 vs. 2.8 %).

**Table 1:** Study population: patients with first myocardial infarction without prior atherosclerotic cardiovascular disease

	All n = 1622	Men n = 1020	Women n = 602
Gender, %	–	63	37
Age, year	68.0 (14.1)	65.4 (13.2)	72.3 (14.4)
Smoking, %	38	42	32
Total cholesterol, mmol/L	5.1 (1.2)	5.0 (1.1)	5.3 (1.4)
LDL cholesterol, mmol/L	3.2 (1.1)	3.2 (1.0)	3.2 (1.2)
HDL cholesterol, mmol/L	1.3 (0.4)	1.2 (0.4)	1.5 (0.5)
Triglycerides, mmol/L	1.6 (1.2)	1.6 (1.3)	1.5 (1.0)
Systolic blood pressure, mmHg	138 (20.5)	138 (19.9)	138 (21.5)
Hypertension, %	65	61	72
Statin, %	16	15	18
Diabetes, %	14	13	16
SCORE (mean)^a^, %	3.9 (3.2)	4.6 (3.4)	2.8 (2.1)

Baseline characteristics are provided as mean (standard deviation) for continuous values

^a^SCORE was calculated by the low-risk equations published by Conroy et al. [14]. The on-treatment cholesterol concentration was used for calculation of SCORE in those treated with statins

### Prevalence of diabetes in patients with first MI

**Fig. 1:**
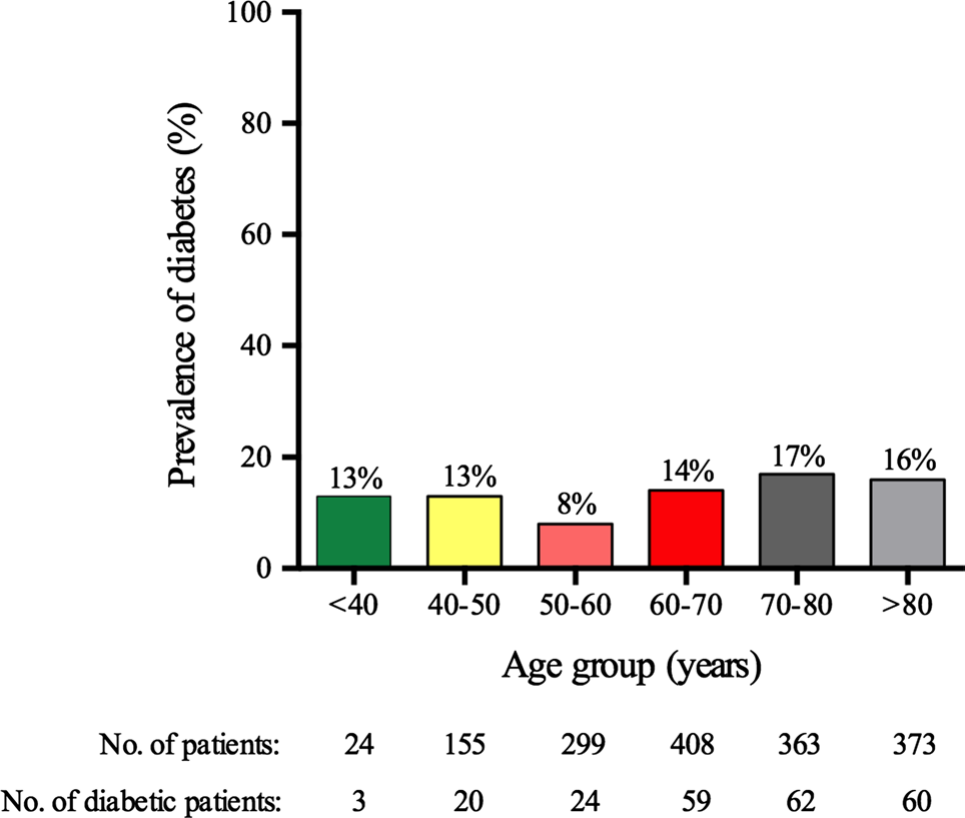
Prevalence of diabetes among patients with first myocardial infarction. The prevalence of known diabetes was similar in all age groups

The prevalence of known diabetes among patients with first MI was 14 % (Table 1), and this prevalence was consistent across different age groups (Fig. 1). All but three of the 228 patients with known diabetes before MI were ≥40 years of age.

Diabetic patients were older (70 vs 68, p = 0.006), were more often women (43 vs 36 %, p = 0.05) and had a higher prevalence of hypertension (79 vs 63 %, p < 0.001) compared to non-diabetic patients with first MI (Table 2), while the prevalence of smoking was equally high (36 vs 39 %, p = 0.34). In contrast, total and LDL cholesterol levels were significantly lower among diabetic patients explained, at least partly, by their higher prevalence of statin use prior to MI. Similar results were observed when stratified by gender (Table 3).

**Table 2:** Study population stratified by known diabetes prior to first myocardial infarction

	Diabetes	No diabetes	p value
	n = 228	n = 1394	
Gender (male), %	57	64	0.05
Age, year	70.3 (13.8)	67.6 (14.1)	0.006
Smoking, %	36	39	0.34
Total cholesterol, mmol/L	4.5 (1.4)	5.2 (1.2)	<0.001
LDL cholesterol, mmol/L	2.5 (1.1)	3.3 (1.1)	<0.001
HDL cholesterol, mmol/L	1.2 (0.4)	1.3 (0.4)	<0.001
Triglycerides, mmol/L	1.9 (1.4)	1.5 (1.1)	<0.001
Systolic blood pressure, mmHg	138 (23)	138 (20)	0.68
Hypertension, %	79	63	<0.001
Statin, %	47	11	<0.001
SCORE (mean)^a^, %	3.8 (2.9)	4.0 (3.2)	0.4

Baseline characteristics are provided as mean (standard deviation) for continuous values

^a^SCORE was calculated by the low-risk equations published by Conroy et al. [14]. The on-treatment cholesterol concentration was used for calculation of SCORE in those treated with statins. SCORE was not created for use in patients with diabetes who are classified as high or very high risk, regardless of SCORE

**Table 3:** Study population stratified by known diabetes and gender prior to first myocardial infarction

	Men			Women		
	Diabetes n = 130	No diabetes n = 890	p value	Diabetes n = 98	No diabetes n = 504	p value
Age, year	68.4 (13.7)	65.0 (13.1)	0.006	72.9 (13.5)	72.2 (14.6)	0.64
Smoking, %	36	43	0.12	35	32	0.49
Total cholesterol, mmol/L	4.3 (1.3)	5.1 (1.1)	<0.001	4.7 (1.4)	5.4 (1.3)	<0.001
LDL cholesterol, mmol/L	2.5 (1.1)	3.3 (1.0)	<0.001	2.6 (1.2)	3.3 (1.2)	<0.001
HDL cholesterol, mmol/L	1.2 (0.4)	1.2 (0.4)	0.17	1.3 (0.4)	1.5 (0.5)	<0.001
Triglycerides, mmol/L	1.8 (1.2)	1.6 (1.3)	0.07	2.0 (1.7)	1.4 (0.8)	<0.001
Systolic blood pressure, mmHg	139 (20.8)	138 (19.7)	0.39	138 (24.9)	138 (20.8)	0.73
Hypertension, %	78	59	<0.001	82	70	0.02
Statin, %	45	10	<0.001	49	12	<0.001
SCORE (mean)^a^, %	4.4 (3.2)	4.6 (3.5)	0.59	2.8 (2.2)	2.8 (2.1)	0.73

Baseline characteristics are provided as mean (standard deviation) for continues values

^a^SCORE was calculated by the low-risk equations published by Conroy et al. [14]. The on-treatment cholesterol concentration was used for calculation of SCORE in those treated with statins. SCORE was not created for use in patients with diabetes who are classified as high or very high risk, regardless of SCORE

### Statin vs. non-statin use in diabetic patients

**Fig. 2:**
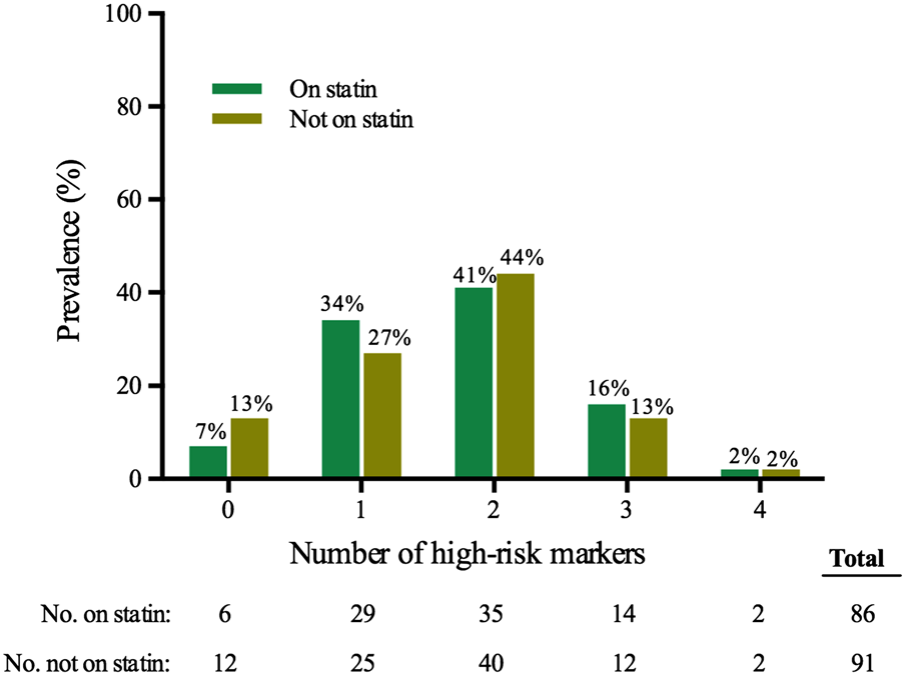
Markers of high cardiovascular risk in diabetic patients with first myocardial infarction, stratified by statin use. The high-risk markers were smoking, hypertension, family history of coronary heart disease, and nephropathy. There was no significant relationship between the number of markers of high or very high risk and statin use prior to a first myocardial infarction [odds ratio of 1.11 (0.81–1.54, p = 0.49) per high-risk marker]. Regardless of statin use, nearly all diabetic patients had at least 1 high-risk marker and the majority had 2 or more high-risk markers prior to myocardial infarction. Analyses were performed among the 177 diabetic patients in whom we had complete information on all markers of high risk, including family history of coronary heart disease

The majority (53 %) of diabetic patients was not using statins before their first MI (Table 4). Many of those not treated with statins before MI had hypertension (71 %) or were current smokers (37 %). Nearly all (~95 %) had at least one and the majority had two or more of the following markers of very high cardiovascular risk: hypertension, smoking, family history of CHD or nephropathy (GFR < 60 mL/min/1.73 m^2^) (Fig. 2).

**Table 4:** Diabetic patients stratified by statin use prior to first myocardial infarction

	On statins before MI	Not on statins before MI	p value
	n = 106	n = 122	
Gender, %	–	–	0.51
Age, year	70.1 (12.0)	70.6 (15.2)	0.80
Smoking, %	34	37	0.21
Total cholesterol, mmol/L	4.1 (1.2)	4.8 (1.4)	<0.001
LDL cholesterol, mmol/L	2.1 (0.9)	2.9 (1.2)	<0.001
HDL cholesterol, mmol/L	1.2 (0.4)	1.2 (0.4)	0.42
Triglycerides, mmol/L	1.8 (1.6)	1.9 (1.3)	0.45
Systolic blood pressure, mmHg	139 (21.5)	138 (24.0)	0.76
Hypertension, %	89	71	0.001
Antihypertensive medicine, %	79	60	0.002
GFR^c^, mL/min/1.73 m^2^	64 (27.3)	72 (27.3)	0.03
Nephropathy (GFR < 60)^c^, %	43	33	0.69
Family history of CHD, %	37	34	0.62
LDL cholesterol ≥2 mmol/L, %	54	81	<0.001
LDL cholesterol ≥1.8 mmol/L, %	57	83	<0.001
SCORE (mean), %	–	3.8 (2.8)	
SCORE (mean) before statin therapy^a^, %	4.6 (3.8)		–
Estimated 10-year risk in diabetes (mean)^b^, %	16.6 (12.4)	13.7 (10.2)	0.10
Estimated 10-year risk in diabetes ≥5 %^b^, n/n	91 %	85 %	0.22

Baseline characteristics are provided as mean (standard deviation) for continuous values

^a^SCORE was calculated after adding 1.5 mmol/L to the measured on-treatment total cholesterol concentration (an estimate of the pre-treatment SCORE risk)

^b^SCORE risk multiplied by 3 in men and 5 in women to take the diabetes-related risk into account [5, 6, 15]

^c^ *GFR* glomerular filtration rate; GFR < 60 mL/min/1.73 m^2^ is defined as moderate-to-severe chronic kidney disease in the ESC guideline [6]

In patients using statins before MI we added 1.5 mmol/L to the measured on-treatment total cholesterol concentration [23] and then calculated the SCORE risk, using this as an estimate for the pre-treatment SCORE risk. To get an estimate of the real risk by taking the diabetes-related risk into account, we multiplied the calculated SCORE risk by 3 in men and 5 in women [5, 6, 15]. The average 10-year risk for fatal CVD was >10 % (very high risk) not only in those on statins before MI (pre-treatment risk) but also in those not on statins (Table 4). A similar proportion of diabetic patients treated or not treated with statins before MI had a real cardiovascular risk higher than the 5 % high-risk threshold defined by the ESC guidelines (91 % vs. 85 %, p = 0.22; Table 4) [4–6]. The 10-year risk for fatal CVD would have been even higher if we instead of the low-risk SCORE equations had used the high-risk equations to estimate risk, which were recommended in Denmark during the study period [15].

### Factors associated with statin use in diabetic patients

The multivariable adjusted association of cardiovascular risk factors with statin use in diabetic patients is shown in Table 5. Total cholesterol and hypertension were associated with taking statins prior to MI, while age, gender and smoking status were not. These results were similar in men and women separately. Surprisingly, increasing number of high-risk markers (hypertension, smoking, family history of CHD and nephropathy) was not significantly associated with statin treatment prior to MI (odds ratio of 1.11 (0.81–1.54, p = 0.49) per high-risk marker) (Fig. 2).

**Table 5:** Association between cardiovascular risk factors and statin use in diabetic patients prior to first myocardial infarction

Risk factor	Odds ratio (95 % CI) for statin use		
	Overall	Men	Women
Age, per 5-year increase	0.97 (0.87–1.09)	1.07 (0.93–1.24)	0.82 (0.68–0.99)
Sex	1.0 (0.55–1.81)	–	–
Current smoking	0.77 (0.41–1.45)	0.83 (0.36–1.95)	0.68 (0.25–1.82)
Total cholesterol^a^	1.51 (1.22–1.87)	1.66 (1.24–2.22)	1.29 (0.93–1.79)
Hypertension	3.36 (1.53–7.35)	3.06 (1.14–8.27)	5.46 (1.35–22.08)

Multivariable adjusted odds ratios with 95 % confidence intervals (CI) calculated using logistic regression

^a^1.5 mmol/L were added to the measured on-treatment total cholesterol concentration in patients taking statins before myocardial infarction

## Discussion

Among consecutive patients with MI as first manifestation of ASCVD, 14 % had known diabetes before MI. Primary prevention with statins had been initiated in 47 and 11 % of those with or without known diabetes, respectively. Nearly all diabetic patients not treated with statins before first MI had at least one marker of very high cardiovascular risk, including hypertension (71 %), smoking (37 %) and nephropathy (33 %). Surprisingly, these markers of very high cardiovascular risk were not more prevalent in diabetic patients treated with statins prior to MI compared with diabetic patients not treated with statins.

### Known diabetes before first MI

With the growing burden of obesity and type 2 diabetes, it may appear surprising that only 14 % of first MI occurred in patients with known diabetes. However, this is in line with 11 % in the Danish DANAMI-2 study in patients with ST-elevation MI [24] and 15 % in a more recent Swiss study in patients with first acute coronary syndrome [25]. In the INTERHEART study, the prevalence of diabetes was 18 % in patients with first MI [26]. A much higher prevalence of diabetes among MI patients have been reported in studies that included patients with both first and recurrent MI and diabetes diagnosed not only before but also after admission [27–29].

### Diabetes-related risk

In the decades-old SCORE cohorts, information on diabetes was not collected uniformly to allow inclusion of diabetes as a predictor in the SCORE model [14]. Instead diabetes type 2 (and type 1 with microalbuminuria) was defined as a high-risk condition with similar targets for cholesterol-lowering treatment as in patients with clinically established ASCVD [4]. In the most recent ESC guidelines, patients with diabetes were classified as high (SCORE 5–10 %) or very-high (SCORE ≥10 %) risk depending on the presence of cardiovascular risk factors and end-organ damage [6]. It was stated that a re-analysis of the SCORE database indicated that diabetes increases the risk for fatal ASCVD ~5 times in women and ~3 times in men [5, 6, 15]. Based on this information we estimates a likely risk in the diabetic patients with first MI and found that most passed the 10 % very high risk threshold set by the ESC guidelines, and nearly all passed the 5 % high risk threshold (Table 4). However, considering the substantial decline in ASCVD mortality since the SCORE data was collected decades ago, the relevance of these estimates may be questioned.

More recent data indicates that the diabetes-related risk is heterogeneous and often lower than that estimated from the SCORE database, depending on the used predictors, predicted outcomes (e.g., fatal vs. non-fatal ASCVD), presence of other cardiovascular risk factors and end-organ damage, and duration of diabetes [30, 31]. Worth to recognize is that a study that contributed significantly to the classification of diabetes as a CHD risk equivalent originated from a high-risk country (Finland in the 1980s), with a high prevalence of hypercholesterolemia and hypertension in the diabetic population, and an average duration of diabetes of 8 years [2, 3]. Today, screen-detected and treated diabetes is associated with a much lower cardiovascular risk [32], but still with a stronger impact of diabetes on ASCVD risk in women than men. Why remains unclear [33].

Much remains to be understood about the diabetes-related risk for ASCVD. It is still uncertain whether hyperglycemia per se accelerates the development of atherothrombosis, and if it does, whether qualitative (plaque vulnerability) or quantitative (plaque burden) changes are most important [34]. Targeting the traditional cardiovascular risk factors such as high blood pressure and cholesterol is at least as important in patients with diabetes as in those without diabetes [35]. Interestingly, statin therapy before a first MI may improve long-term survival in overweight patients with diabetes [36]. Even though concerns have been raised about the diabetogenic effect of statin therapy [37], statins certainly reduce the risk for ASCVD in patients with diabetes.

### Primary prevention with statins in diabetes

Based on evidence from randomized controlled trials [38–40], current guidelines recommend primary prevention with statins to nearly all adults with diabetes [5–11, 17–22]. The strong indication for statin therapy to patients with diabetes, especially in the presence of ≥1 cardiovascular risk factor and/or end-organ damage, is not new and was effective during the present study period [17, 18]. The European (and Danish) guidelines recommend therapeutic goals, but we focused primarily on initiation of statin therapy rather than on treatment goal because it is more important to initiate therapy than to intensify it to fulfil a treat-to-target strategy [41]. Therefore, it came as a surprise that statin therapy had been initiated in less than half of patients with known diabetes before first MI, in particular because nearly all had at least one easily identifiable marker of very high risk, such as hypertension, smoking, family history of CHD, and/or nephropathy. Thus, the current practice of selective use of statins for the primary prevention of ASCVD in some but not other patients with diabetes does not seem to target treatment to those at highest risk. The same appears to be the case in non-diabetic patients destined for a first MI [42].

Our data do not explain why so many diabetic patients at very high cardiovascular risk are not treated with statins. Denmark has a universal healthcare system that covers all citizens. Visits to general practitioners and specialists are free of charge, and diabetic patients are followed regularly by their general practitioner and/or diabetes outpatient clinics. The European guidelines on ASCVD prevention classified diabetes as a high-risk condition more than 10 years ago [4]. These guidelines were endorsed by Danish medical societies including, among others, the Danish Society of Cardiology, Danish Endocrine Society, and the Danish College of General Practitioners, and the importance of primary prevention with statins to diabetic patients at high risk has been stressed for many years [17–22]. However, the adherence to guideline recommendations is often low in general practice [43]. In a recent primary care study, only 45 % of patients with known type 2 diabetes were treated with a statin or another lipid-lowering drug [43]. The proportion of patients with screen-detected diabetes treated with statins varied widely between general practices, from 0 to 100 % [44]. In the ADDITION trial, in which general practitioners voluntarily had agreed to compare routine care of screen-detected diabetes with intensive treatment of multiple risk factors, the adherence to the given recommendations was low [44], which might have contributed to the neutral results [32]. Although patients differ in their willingness and ability to comply with prescribed medication, the great variability among general practitioners in the use of statins in patients diagnosed with diabetes indicates that primary care providers play a critical role in optimal implementation of the guidelines.

### Smoking in diabetes

Active smoking increases the risk of type 2 diabetes, amplifies the diabetes-related risk for ASCVD, and accelerates the progression of diabetic nephropathy [45–47]. In Denmark, 10–15 % of the general population were current smokers in 2010–2012 [48]. In the present study, more than one-third of both the diabetic (36 %) and the non-diabetic (39 %) patients with first MI were current smokers, confirming the strong impact of smoking on cardiovascular risk. The recommendation to stop smoking is unanimously strong [7, 11, 17–19] even though the glycemic control may deteriorate temporarily after cessation of smoking [49]. The high prevalence of smoking in diabetic patients at very high risk for ASCVD indicates that current guidelines are insufficiently implemented.

### Strengths and limitations

Our study has several important strengths. The study population consists of contemporary and consecutive (unselected) real-world patients with a first MI (hard ASCVD endpoint). Thus, the age, sex and risk factor distribution is representative of that seen in daily clinical practice of patients with first MI. Statin use before MI was assessed in patients who knew they had diabetes and were treated with diet and/or anti-diabetic drugs before the acute event.

A limitation is the retrospective nature of the study. Potential limitations of risk factor assessment after versus before MI is not pertinent to the key message of the present study and has been discussed previously [13]. We may have underestimated the cardiovascular risk prior to statin therapy just by adding 1.5 mmol/L to the total cholesterol concentration, because people who decide to take statins may also try to improve other risk factors than cholesterol.

## Conclusion

Approximately 14 % of 1622 consecutive patients hospitalized with a first MI had known diabetes. Primary prevention with statins had been initiated in less than half of the diabetic patients, although nearly all had one or more markers of very high cardiovascular risk. These results highlight an urgent need for optimizing statin therapy and global risk factor control in diabetic patients without known ASCVD.

## Abbreviations

ASCVD: atherosclerotic cardiovascular disease
CHD: coronary heart disease
CVD: cardiovascular disease
ESC: European Society of Cardiology
GFR: glomerular filtration rate
HDL-C: high-density lipoprotein cholesterol
LDL-C: low-density lipoprotein cholesterol
MI: myocardial infarction
NCEP: National Cholesterol Education Program
SCORE: systematic coronary risk evaluation

## References

[1] Kannel WB, McGee DL (1979). Diabetes and cardiovascular disease. The Framingham study. JAMA.

[2] Haffner SM, Lehto S, Rönnemaa T, Pyörälä K, Laakso M (1998). Mortality from coronary heart disease in subjects with type 2 diabetes and in nondiabetic subjects with and without prior myocardial infarction. N Engl J Med.

[3] Expert Panel on Detection (2001). Evaluation, and treatment of high blood cholesterol in adults. Executive summary of the third report of the national cholesterol education program (NCEP) expert panel on detection, evaluation, and treatment of high blood cholesterol in adults (adult treatment panel III). JAMA.

[4] De Backer G, Ambrosioni E, Borch-Johnsen K, Brotons C, Cifkova R, Dallongeville J (2003). European guidelines on cardiovascular disease prevention in clinical practice. Eur Heart J.

[5] Reiner Z, Catapano AL, De Backer G, Graham I, Taskinen MR, Wiklund O (2011). ESC/EAS guidelines for the management of dyslipidaemias. Eur Heart J.

[6] Perk J, De Backer G, Gohlke H, Graham I, Reiner Z, Verschuren M (2012). European guidelines on cardiovascular disease prevention in clinical practice (version 2012). Eur Heart J.

[7] Rydén L, Grant PJ, Anker SD, Berne C, Cosentino F, Danchin N (2013). ESC guidelines on diabetes, pre-diabetes, and cardiovascular diseases developed in collaboration with the EASD: the task force on diabetes, pre-diabetes, and cardiovascular diseases of the European Society of Cardiology (ESC) and developed in collaboration with the European Association for the Study of Diabetes (EASD). Eur Heart J.

[8] Stone NJ, Robinson J, Lichtenstein AH, Bairey Merz CN, Lloyd-Jones DM, Blum CB (2013). ACC/AHA guideline on the treatment of blood cholesterol to reduce atherosclerotic cardiovascular risk in adults: a report of the American College of Cardiology/American Heart Association Task Force on Practice Guidelines. J Am Coll Cardiol.

[9] JBS3 Board (2014). Joint British Societies’ consensus recommendations for the prevention of cardiovascular disease (JBS3). Heart..

[10] National Institute for Health and Care Excellence (NICE). Clinical guideline CG181: lipid modification—cardiovascular risk assessment and the modification of blood lipids for the primary and secondary prevention of cardiovascular disease. National Clinical Guideline Centre, 2014. http://www.nice.org.uk/Guidance/cg181. Accessed 26 July 2014. 25340243

[11] Association The American Diabetes (2015). Standards of medical care in diabetes—2015: summary of revisions. Diabetes Care.

[12] Thygesen K, Alpert JS, White HD (2007). Joint ESC/ACCF/AHA/WHF task force for the redefinition of myocardial infarction. Universal definition of myocardial infarction. Eur Heart J.

[13] Mortensen MB, Falk E (2014). Real-life evaluation of European and American high-risk strategies for primary prevention of cardiovascular disease in patients with first myocardial infarction. BMJ Open.

[14] Conroy RM, Pyörälä K, Fitzgerald AP, Sans S, Menotti A, De Backer G (2003). Estimation of ten-year risk of fatal cardiovascular disease in Europe: the SCORE project. Eur Heart J.

[15] Graham I, Atar D, Borch-Johnsen K, Boysen G, Burell G, Cifkova R (2007). European guidelines on cardiovascular disease prevention in clinical practice: executive summary: fourth joint task force of the European society of cardiology and other societies on cardiovascular disease prevention in clinical practice. Eur Heart J.

[16] ESC HeartScore risk calculator. http://www.heartscore.org. Accessed 21 May 2016.

[17] Danish Society of Cardiology and Danish Endocrine Society. Guideline on diabetes and heart disease (in Danish). Danish Society of Cardiology. 2008.

[18] Danish College of General Practitioners, Danish Endocrine Society, and Institute for Rational Pharmacotherapy. Guidelines for type 2 diabetes: a joint treatment guideline with identical clinical treatment goals (in Danish). 2011.

[19] Danish Endocrine Society and Danish College of General Practitioners. Pharmacological treatment of type 2 diabetes: 2014 revision of guidelines for type 2 diabetes (in Danish). 2014.

[20] European Guidelines on cardiovascular disease prevention in clinical practice (version 2012) endorsed by the Danish Society of Cardiology, Jan 2013. Cardiologisk Forum, Feb 2013.

[21] Danish Endocrine Society. National treatment guideline, endocrinology: treatment and control of type 2 diabetes. National BehandlingsVejledning i Endokrinologi. 2014.

[22] Danish Society of Cardiology. National treatment guideline, cardiology: diabetes and heart disease (National BehandlingsVejledning 2015). 2015.

[23] Naci H, Brugts JJ, Fleurence R, Ades AE (2013). Dose-comparative effects of different statins on serum lipid levels: a network meta-analysis of 256,827 individuals in 181 randomized controlled trials. Eur J Prev Cardiol.

[24] Madsen MM, Busk M, Søndergaard HM, Bøttcher M, Mortensen LS, Andersen HR (2005). DANAMI-2 investigators. Does diabetes mellitus abolish the beneficial effect of primary coronary angioplasty on long-term risk of reinfarction after acute ST-segment elevation myocardial infarction compared with fibrinolysis? (A. DANAMI-2 substudy). Am J Cardiol.

[25] Selby K, Nanchen D, Auer R, Gencer B, Räber L, Klingenberg R (2015). Low statin use in adults hospitalized with acute coronary syndrome. Prev Med.

[26] Yusuf S, Hawken S, Ounpuu S, Dans T, Avezum A, Lanas F (2004). INTERHEART study investigators. Effect of potentially modifiable risk factors associated with myocardial infarction in 52 countries (the INTERHEART study): case-control study. Lancet.

[27] Norhammar A, Malmberg K, Rydén L, Tornvall P, Stenestrand U, Wallentin L (2003). Register of information and knowledge about Swedish heart intensive care admission (RIKS-HIA). Under utilisation of evidence-based treatment partially explains for the unfavourable prognosis in diabetic patients with acute myocardial infarction. Eur Heart J.

[28] Gore MO, Patel MJ, Kosiborod M, Parsons LS, Khera A, de Lemos JA, Rogers WJ, Peterson ED, Canto JC, McGuire DK (2012). Diabetes mellitus and trends in hospital survival after myocardial infarction, 1994 to 2006: data from the national registry of myocardial infarction. Circ Cardiovasc Qual Outcomes.

[29] Ahmed B, Davis HT, Laskey WK (2014). In-hospital mortality among patients with type 2 diabetes mellitus and acute myocardial infarction: results from the national inpatient sample, 2000–2010. J Am Heart Assoc.

[30] Sattar N (2013). Revisiting the links between glycaemia, diabetes and cardiovascular disease. Diabetologia.

[31] Collaboration Emerging Risk Factors (2010). Diabetes mellitus, fasting blood glucose concentration, and risk of vascular disease: a collaborative meta-analysis of 102 prospective studies. Lancet.

[32] Griffin SJ, Borch-Johnsen K, Davies MJ, Khunti K, Rutten GE, Sandbæk A (2011). Effect of early intensive multifactorial therapy on 5-year cardiovascular outcomes in individuals with type 2 diabetes detected by screening (ADDITION-Europe): a cluster-randomised trial. Lancet.

[33] Peters SA, Huxley RR, Sattar N, Woodward M (2015). Sex differences in the excess risk of cardiovascular diseases associated with type 2 diabetes: potential explanations and clinical implications. Curr Cardiovasc Risk Rep..

[34] Duce SL, Weir-McCall JR, Gandy SJ, Matthew SZ, Cassidy DB, McCormick L, Rauchhaus P, Looker H, Colhoun HM, Houston JG (2015). Cohort comparison study of cardiac disease and atherosclerotic burden in type 2 diabetic adults using whole body cardiovascular magnetic resonance imaging. Cardiovasc Diabetol.

[35] Wilke T, Mueller S, Groth A, Fuchs A, Seitz L, Kienhöfer J, Maywald U, Lundershausen R, Wehling M (2015). Treatment-dependent and treatment-independent risk factors associated with the risk of diabetes-related events: a retrospective analysis based on 229,042 patients with type 2 diabetes mellitus. Cardiovasc Diabetol.

[36] Colombo MG, Meisinger C, Amann U, Heier M, von Scheidt W, Kuch B, Peters A, Kirchberger I (2015). Association of obesity and long-term mortality in patients with acute myocardial infarction with and without diabetes mellitus: results from the MONICA/KORA myocardial infarction registry. Cardiovasc Diabetol.

[37] Mansi IA, English J, Zhang S, Mortensen EM, Halm EA. Long-term outcomes of short-term statin use in healthy adults: a retrospective cohort study. Drug Saf. 2016. **(Epub ahead of print)**. 10.1007/s40264-016-0412-2 26979831

[38] Collins R, Armitage J, Parish S, Sleigh P, Peto R (2003). Heart protection study collaborative group. MRC/BHF heart protection study of cholesterol-lowering with simvastatin in 5963 people with diabetes: a randomised placebo-controlled trial. Lancet.

[39] Colhoun HM, Betteridge DJ, Durrington PN, Hitman GA, Neil HA, Livingstone SJ, Thomason MJ, Mackness MI, Charlton-Menys V, Fuller JH (2004). CARDS investigators. Primary prevention of cardiovascular disease with atorvastatin in type 2 diabetes in the collaborative atorvastatin diabetes study (CARDS): multicentre randomised placebo-controlled trial. Lancet.

[40] Kearney PM, Blackwell L, Collins R (2008). Efficacy of cholesterol-lowering therapy in 18,686 people with diabetes in 14 randomised trials of statins: a meta-analysis. Lancet.

[41] Timbie JW, Hayward RA, Vijan S (2010). Variation in the net benefit of aggressive cardiovascular risk factor control across the US population of patients with diabetes mellitus. Arch Intern Med.

[42] Kulenovic I, Mortensen MB, Bertelsen J, May O, Dodt KK, Kanstrup H, Falk E (2016). Statin use prior to first myocardial infarction in contemporary patients: inefficient and not gender equitable. Prev Med.

[43] Guldberg TL, Vedsted P, Kristensen JK, Lauritzen T (2011). Improved quality of type 2 diabetes care following electronic feedback of treatment status to general practitioners: a cluster randomized controlled trial. Diabet Med.

[44] Simmons RK, Carlsen AH, Griffin SJ, Charles M, Christiansen JS, Borch-Johnsen K (2014). Variation in prescribing of lipid-lowering medication in primary care is associated with incidence of cardiovascular disease and all-cause mortality in people with screen-detected diabetes: findings from the ADDITION-Denmark trial. Diabet Med.

[45] Willi C, Bodenmann P, Ghali WA, Faris PD, Cornuz J (2007). Active smoking and the risk of type 2 diabetes: a systematic review and metaanalysis. JAMA.

[46] Qin R, Chen T, Lou Q, Yu D (2013). Excess risk of mortality and cardiovascular events associated with smoking among patients with diabetes: meta-analysis of observational prospective studies. Int J Cardiol.

[47] Chakkarwar VA (2012). Smoking in diabetic nephropathy: sparks in the fuel tank?. World J Diabetes.

[48] Mortensen MB, Afzal S, Nordestgaard BG, Falk E (2015). The high-density lipoprotein-adjusted SCORE model worsens SCORE-based risk classification in a contemporary population of 30,824 Europeans: the Copenhagen general population study. Eur Heart J.

[49] Lycett D, Nichols L, Ryan R, Farley A, Roalfe A, Mohammed MA (2015). The association between smoking cessation and glycaemic control in patients with type 2 diabetes: a THIN database cohort study. Lancet Diabetes Endocrinol.

